# Supplementary material for: Reconstitution of circadian clock in synthetic cells reveals principles of timekeeping
Source: Nat Commun. 2025 Jul 21;16:6686. doi: 10.1038/s41467-025-61844-5 (PMC12280161; doi:10.1038/s41467-025-61844-5)
Supplement: Supplementary file 1 — Supplementary Information [file 41467_2025_61844_MOESM1_ESM.pdf]

Supplementary Information for

Reconstitution of circadian clock in synthetic cells reveals principles of timekeeping

Alexander Zhan Tu Li[1], Andy LiWang[2], Anand Bala Subramaniam[1,*]

[1] Department of Bioengineering, University of California, Merced, CA 95343, [2] Department of Chemistry and Biochemistry, University of California, Merced, CA 95343.

[*] Email: asubramaniam@ucmerced.edu

This Supplementary Information includes Supplementary Figures 1 to 10 and Supplementary Tables 1 to 3

Supplementary Figures

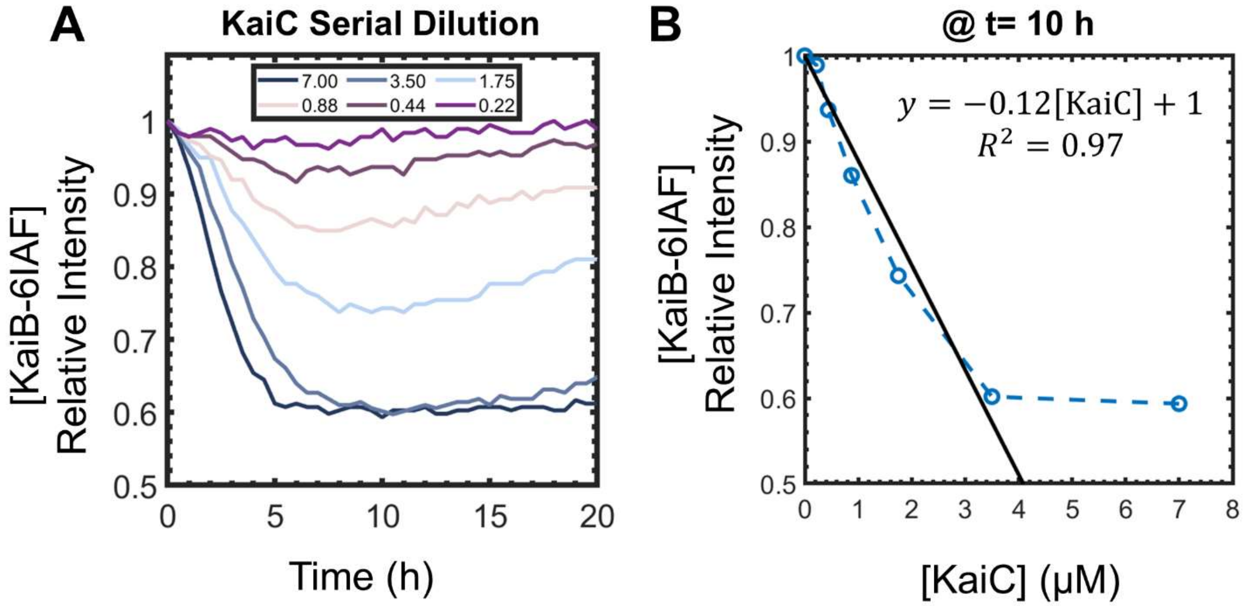

Supplementary Fig. 1 | KaiB-6IAF intensity is quenched upon the binding of KaiC. (A) Kinetic curves of the fluorescence intensity of KaiB-6IAF over 20 hours (h). The concentration of KaiB:KaiB-6IAF (50:50 mol%) was kept constant at 3.5 μM. The concentration of KaiC varied from 7.0 μM to 0.22 μM (various colors). The temperature was maintained at 30 °C. **(B)** Normalized intensities at t = 10 hours plotted against the concentration of KaiC. The open circles are the data points, and the dotted lines are guides to the eye. The solid black line is the result of a linear regression excluding 7.0 μM.

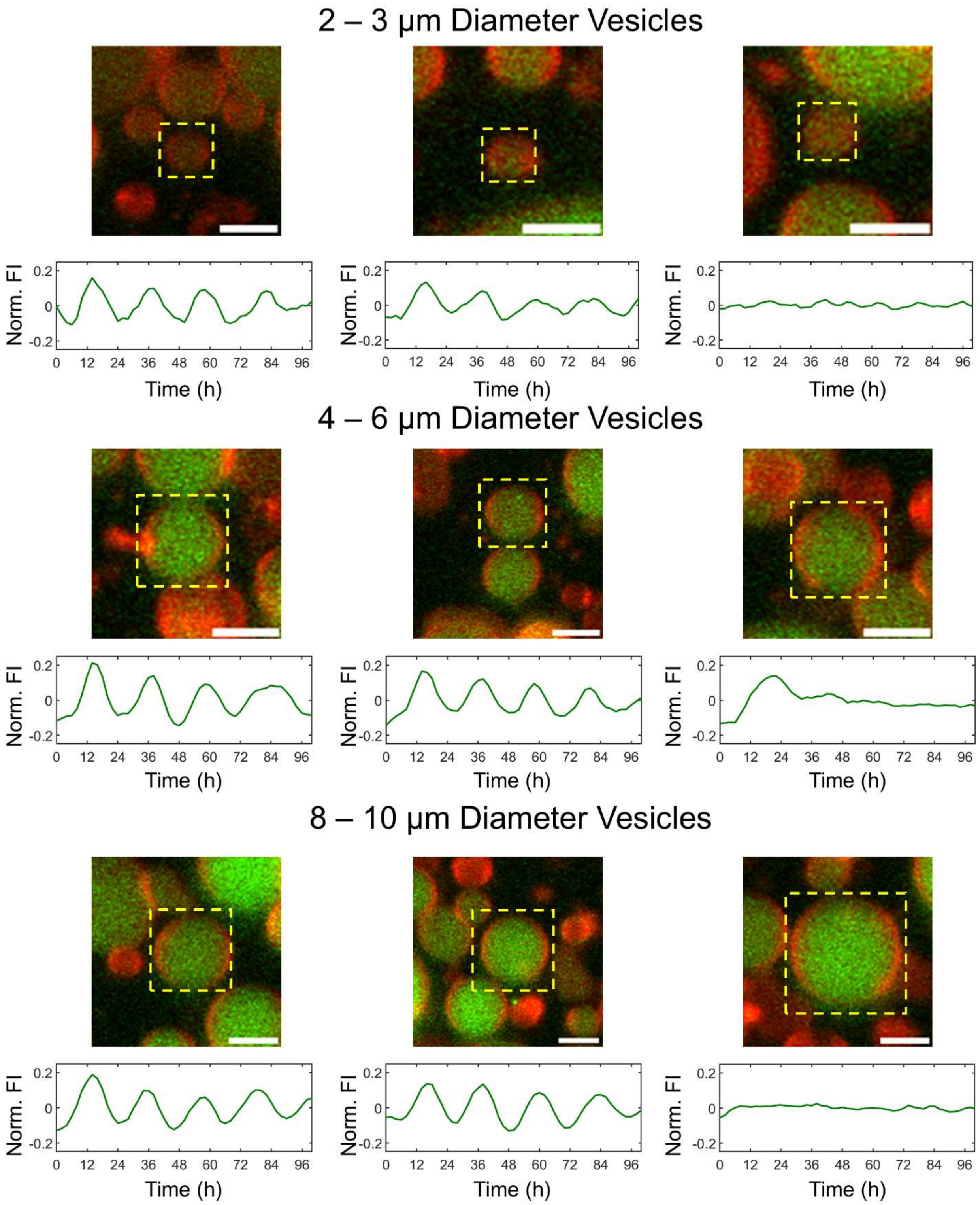

Supplementary Fig. 2 | Clock signals across various PTO-GUV sizes. Static confocal images of PTO-GUVs prepared with 2.5× PTO loading concentration. The yellow dotted box encloses the

PTO-GUV being measured. Corresponding plots of the normalized fluorescence intensity (FI) as a function of time are shown below each image. Scale bars = 5 μm.

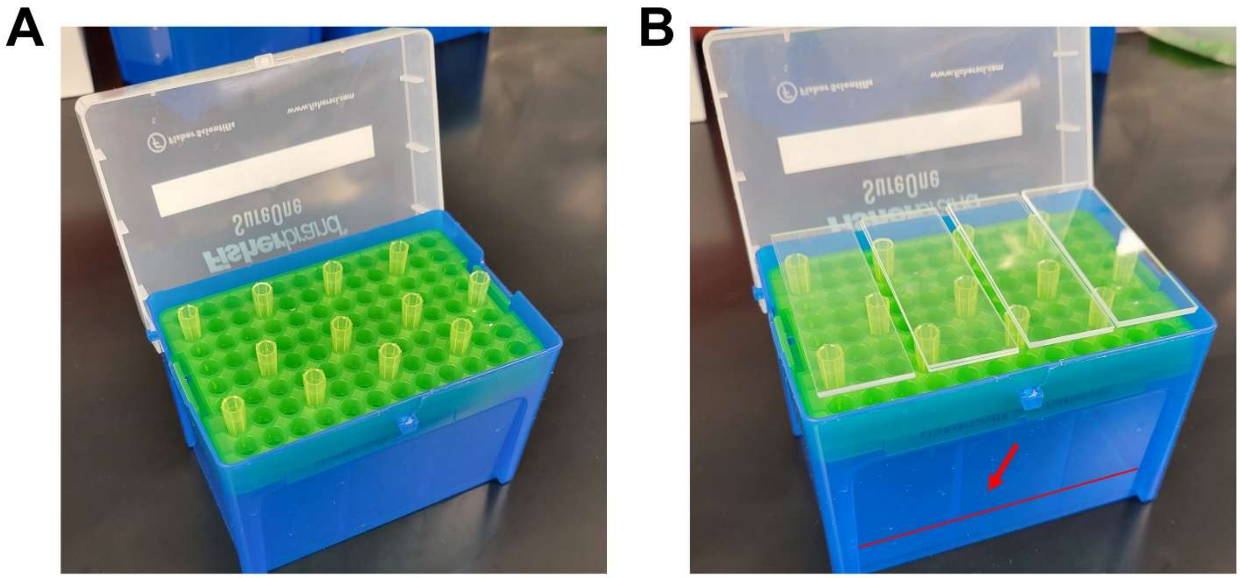

Supplementary Fig. 3 | Humidity chamber for slide functionalization. (A) Arrangement of empty pipette tips in a pipette box to store glass during glass functionalization. **(B)** Two layers of glass sets with the PEG-Biotin functionalization solution sandwiched in between them. The red arrow and line indicate the fill line for water.

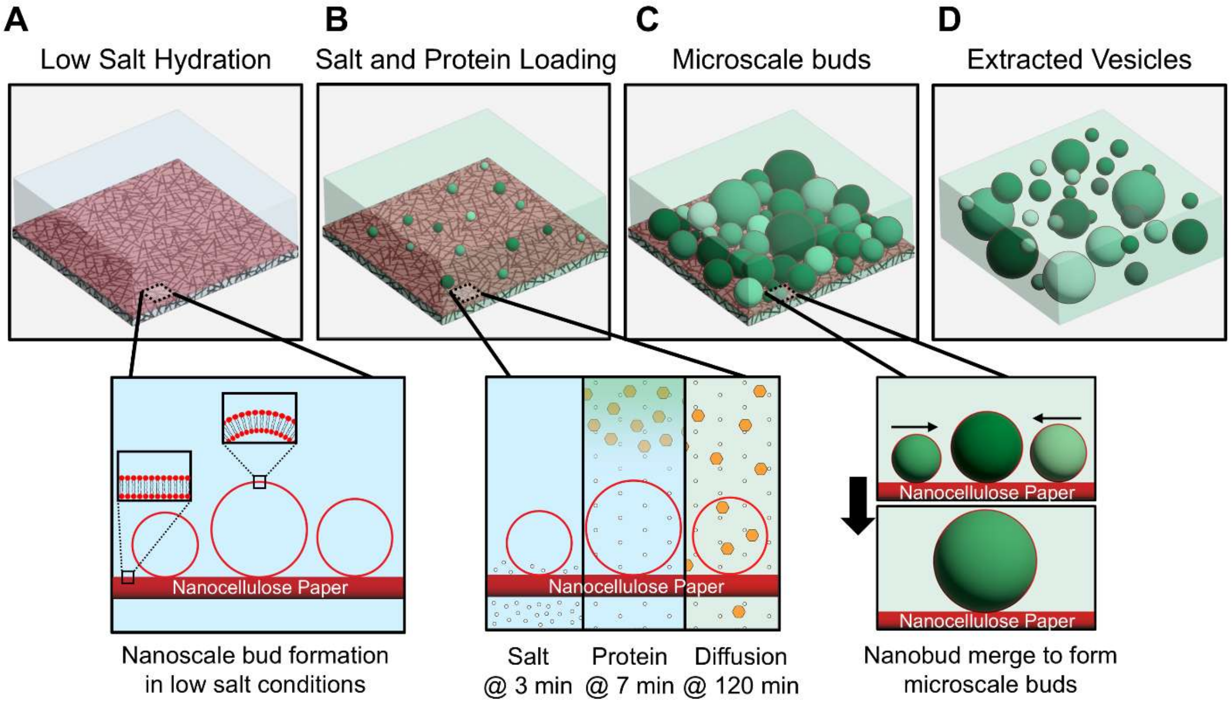

Supplementary Fig. 4 | Assembly of giant unilamellar vesicles containing protein and salt via PAPYRUS-wDL. (A) Nanoscale buds form in low salt conditions for 3 minutes. **(B)** Then concentrated salt (white circles) buffer is added to the external phase beneath the paper. Proteins are added at t = 7 minutes to the external phase and the sample is incubated for a total of 120 minutes. **(C)** Micro- and nano-sized buds merge into large buds during the assembly and loading process. **(D)** The buds are harvested from the surface using a pipette. The buds self-close to form GUVs that trap the protein in their lumens. Variations in protein concentrations between vesicles are indicated by shades of green. Schematics are not to scale.

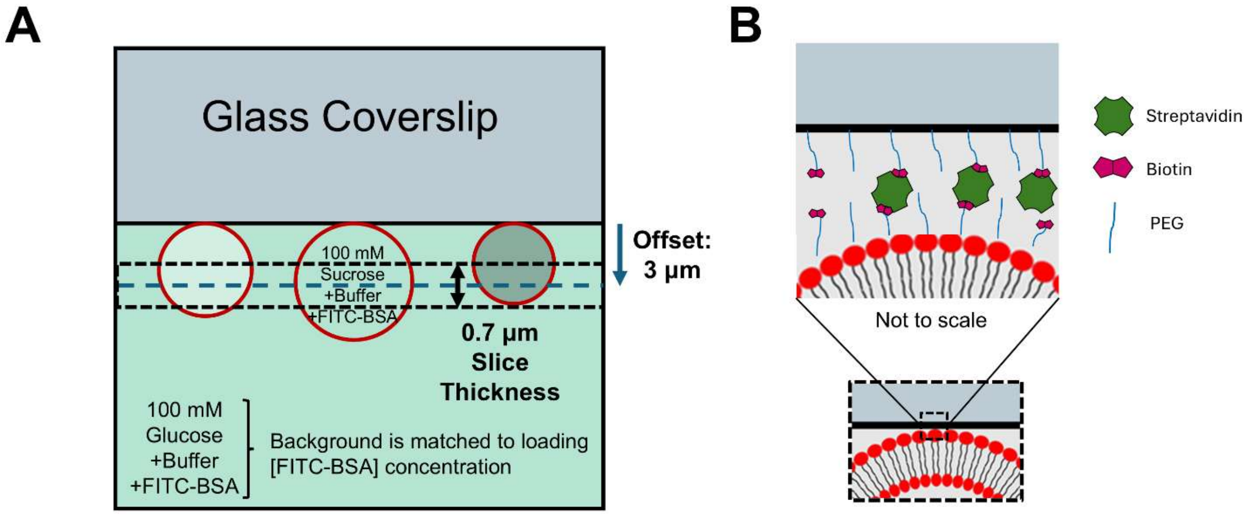

Supplementary Fig. 5 | Schematic of imaging setup for GUVs loaded with FITC-BSA. (A) The focal plane was offset by 3 µm from the surface of the glass coverslip. We used a 63× objective with a numerical aperture of 1.4. The pinhole was set to 1 Airy unit so that the slice thickness was 0.7 µm. The imaging slice will be fully in the lumens of GUVs with diameters ≥ 4 µm. The background had a FITC-BSA concentration that was matched with the loading concentration. (B) Streptavidin-biotin binding was used to tether the vesicles to the surface of the biotin-functionalized glass coverslip. Note that protein and lipid sizes are not drawn to scale in this schematic.

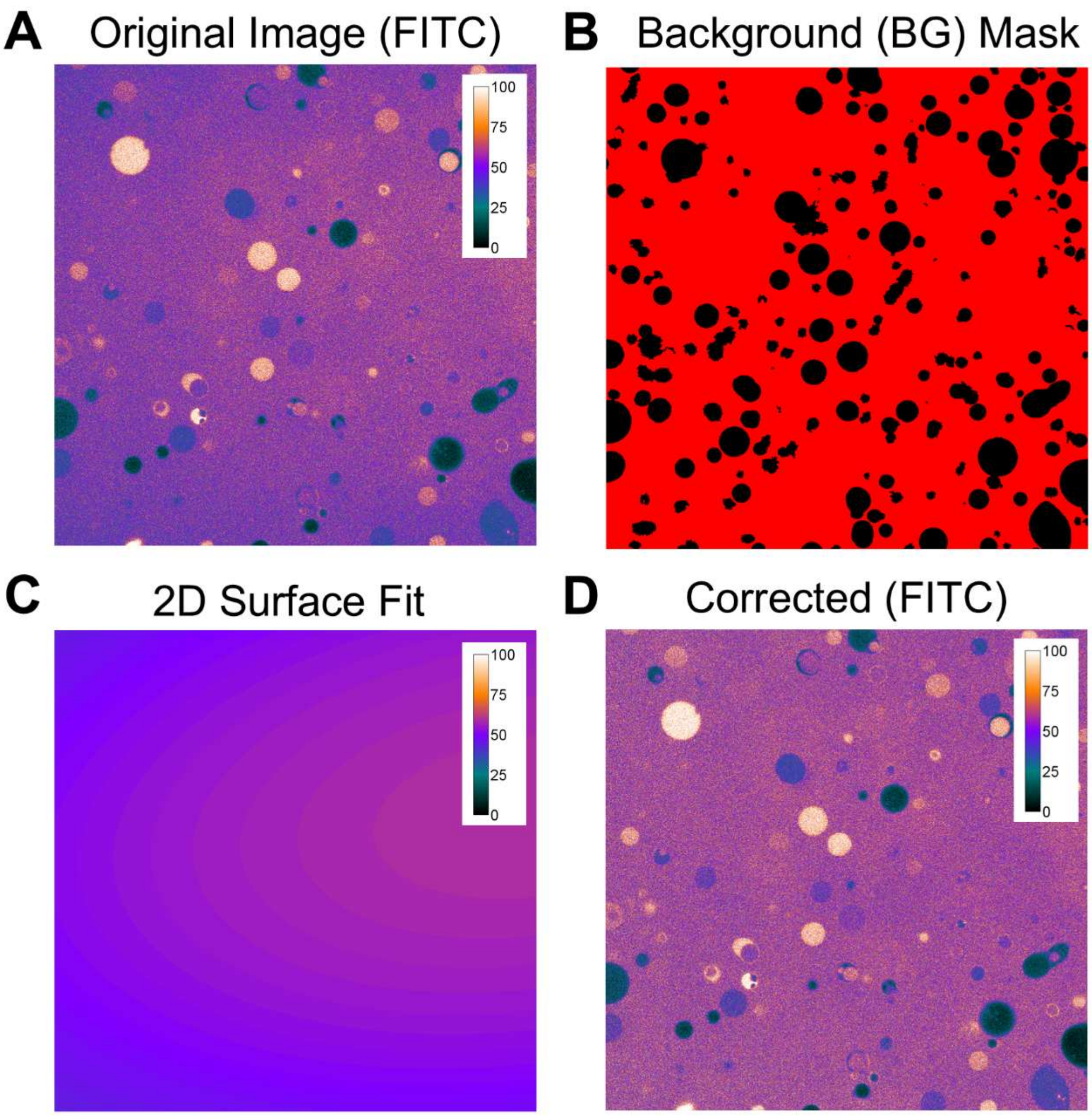

Supplementary Fig. 6 | Example of a 2D polynomial surface fitting algorithm for field-flatness correction. (**A)** Image with uneven intensity. (**B)** Background mask obtained by segmenting the red channel. The background is shown in red, and the excluded objects are black. (**C)** The results of the 2D polynomial surface fit show the correction matrix. (**D)** The corrected image. The color map shows the intensity values.

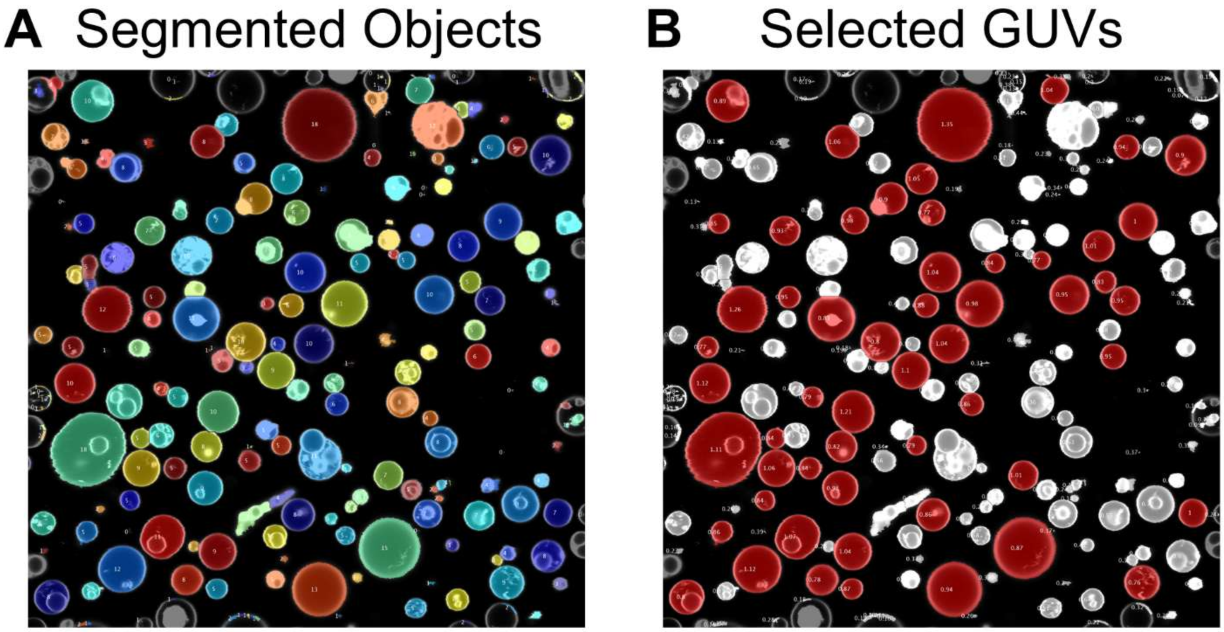

Supplementary Fig. 7 | Example of GUV segmentation. (A) The colored circles are all the fluorescent objects detected by the segmentation routine. Objects touching the edges of the image are excluded. (**B)** A coefficient of variation (CV) based selection algorithm selects GUVs (red) and excludes other objects. The image size was 134.95×134.95 µm (1584×1584 pixels).

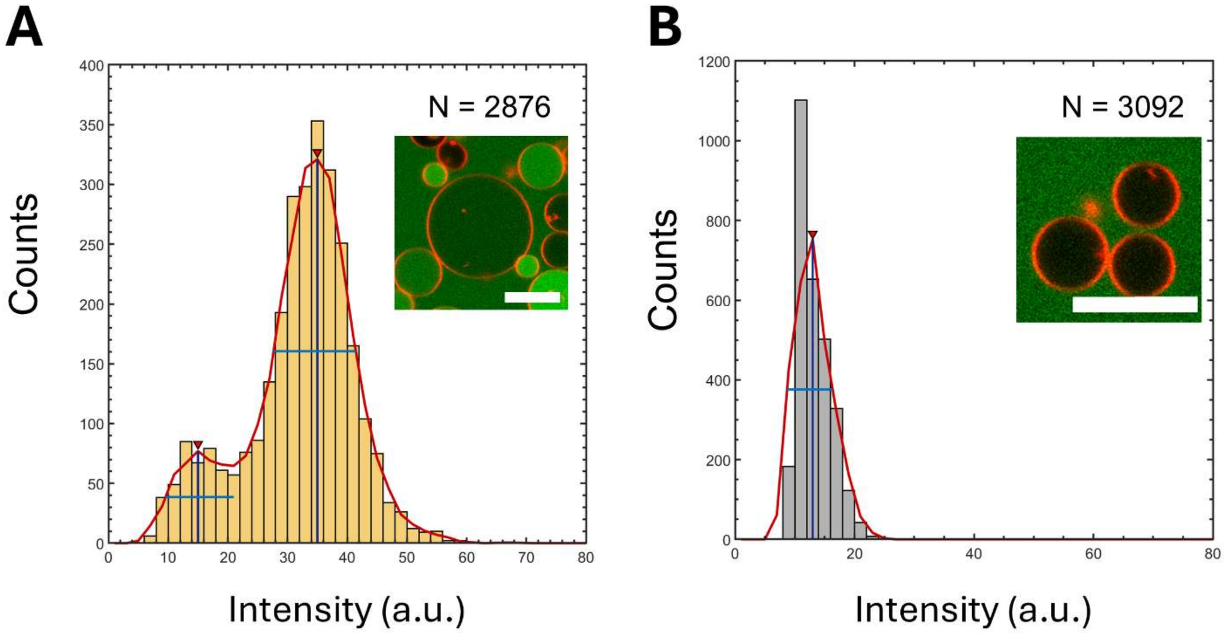

Supplementary Fig. 8 | Analysis of the distribution of intensities. (A) GUVs assembled using PAPYRUS-wDL with an intended loading concentration of 0.88 μM FITC-BSA, showing two peaks, a lower peak corresponding to the empty GUVs and a second peak corresponding to the GUVs with protein **(B)** Empty GUVs, imaged similarly to GUVs assembled using PAPYRUS-wDL, show the fluorescence in the green channel matches the lower intensity peak location and width (FWHM**)** of the empty vesicles in (A). Scale bars = 10 μm.

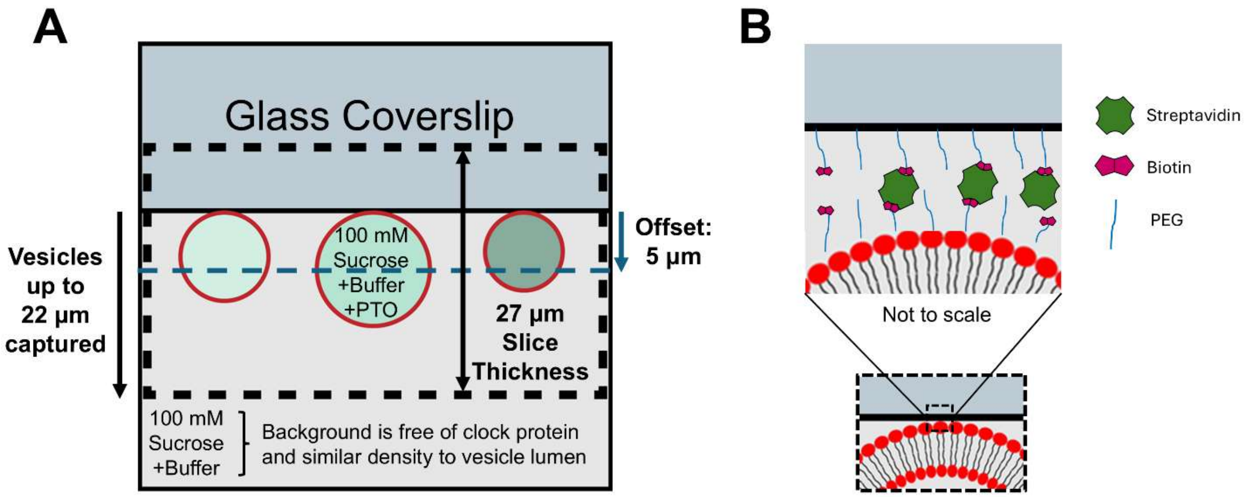

Supplementary Fig. 9 | Schematic of the setup for time-lapse imaging of PTO-GUVs. (A) The focal plane was offset 5 µm from the surface of the glass coverslip. We used a 20× objective with a numerical aperture of 0.8 and opened the pinhole to its maximum diameter. The slice thickness was 27 µm. Thus, vesicles up to 22 µm in diameter were expected to be fully captured in the image. The background was free of clock protein and had a similar density to the vesicle lumen. (B) Streptavidin-biotin binding was used to tether the vesicles to the surface of the biotin-functionalized glass coverslip. Protein and lipids sizes are not drawn to scale in this schematic. Note that this setup differs from Supplementary Fig. 5 in that all vesicles up to 22 µm in diameter are captured within the slice region.

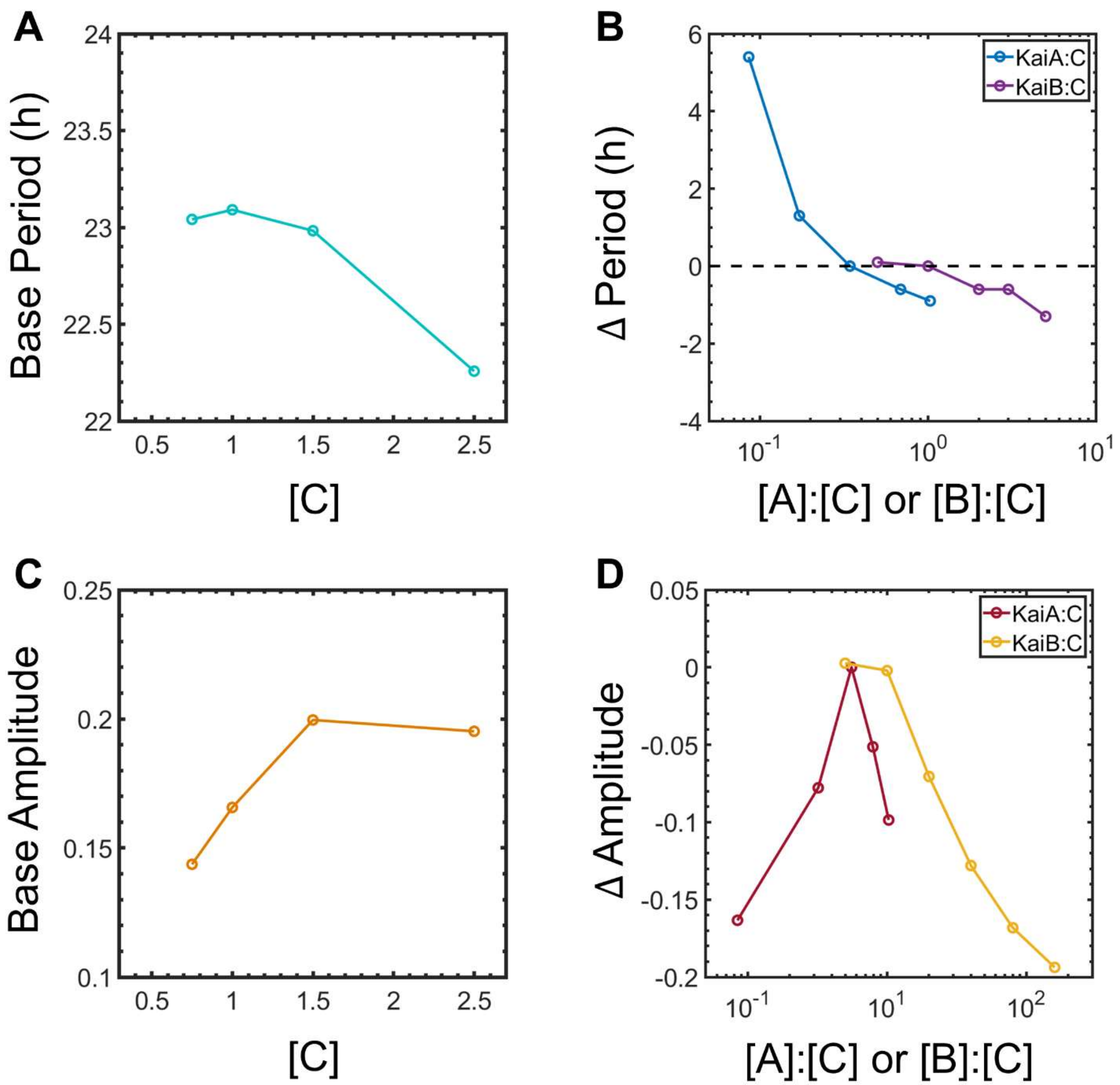

Supplementary Fig. 10 | Empirical relationships between the concentration and stoichiometry of PTO proteins used to obtain periods and amplitudes. (A) Plot of periods vs KaiC concentrations at a fixed 0.34:1:1 KaiA:KaiB:KaiC ratio obtained from bulk plate reader measurements. **(B)** Change in period (ΔPeriod) for different ratios of KaiA:KaiC and KaiB:KaiC. The data was adapted from [8]**Click or tap here to enter text.** to calculate the change in period due to changes in KaiA:C and KaiB:C ratios. **(C)** Plot of amplitude vs KaiC concentration at a fixed

0.34:1:1 KaiA:KaiB:KaiC ratio obtained from bulk plate reader measurements. **(D)** Change in amplitude (ΔAmplitude) for different ratios of KaiA:KaiC and KaiB:KaiC. The relationship of the amplitude with KaiB:C ratio was obtained from Supplementary Fig. 1B.

Supplementary Tables

**Step**	**Name**	**Concentration**	**Volume**	**Addition Time**
Budding	Sucrose	119 mM	126 μL	0 min
Salt	Clock buffer	10×	14 μL	3 min
Protein	FITC-BSA (1 × clock buffer)	15×	10 μL	10 min
**End**	Final Composition	100 mM sucrose, 1× clock buffer, 1× FITC-BSA	150 μL	

Supplementary Table 1 Table of additions for diffusive loading. The total incubation time from start to finish is 120 min.

Source	SS	df	MS	F	Prob > F (p-value)	Comments
Columns	0.001909	3	0.00636	1.19	0.3734	Not significant
Error	0.0428	8	0.00535			
Total	0.06189	11				

Supplementary Table 2 ANOVA table comparing the significance of the CV values obtained from N = 3 independent repeats of groups with loading concentrations of 4.5, 2.63, 1.75, and 0.88 μM of FITC-BSA (groups). There is no significant difference in the value of the CV.

Source	SS	df	MS	F	Prob > F (p-value)	Comments
Columns	0.00443	3	0.00148	0.46	0.7159	Not significant
Error	0.02553	8	0.00319			
Total	0.02997	11				

Supplementary Table 3 ANOVA table comparing the significance of the empty vesicle fraction obtained from N = 3 independent repeats of groups with loading concentrations of 4.5, 2.63, 1.75, and 0.88 μM of FITC-BSA (groups). There is no significant difference in the value of the empty fraction.
